# Barriers to the Cervical Cancer Screening by CPC-28 Questionnaire: A Pilot Study

**DOI:** 10.26502/acbr.50170289

**Published:** 2022-09-20

**Authors:** Viera Svihrova, Lukas Kocsis, Jan Svihra, Veronika Szaboova

**Affiliations:** 1 Department of Public Health, Jessenius Faculty of Medicine in Martin, Comenius University Bratislava, Mala Hora 11149/4B, 036 01 Martin, Slovakia; 2 Onkomed ZA, Vojtecha Spanyola 8685, 010 01 Zilina, Slovakia; 3 Department of Urology, University Hospital Martin, Kollarova 2, 036 01 Martin, Slovakia; 4 Medirex, a. s., Novozamocka 67, 949 05 Nitra, Slovakia

**Keywords:** Barriers, Cervical Cancer, CPC-28 Questionnaire, Screening

## Abstract

**Introduction:**

The aim of this study was to identify and compare barriers to cervical cancer screening (CCS) between women seeking and not seeking CCS by CPC-28 questionnaire (‘Creencias, Papanicolaou, Cancer-28’ questionnaire – Beliefs about Papanicolaou and Cervical Cancer).

**Methods:**

A pilot study was performed in 20 gynecological departments, each department sending data from five healthy women and five untreated women with cervical cancer. The women completed a validated and standardized questionnaire with 28 statements (the CPC-28 questionnaire). The participants were divided into women not seeking CCS (8 healthy women vs 30 women with cervical cancer) and women seeking CCS (54 healthy women vs 43 women with cervical cancer). A four-point Likert scale (item score from 1 to 4) was used to assess responses. A linear transformation was made to calculate the responses. Differences with a p value of < 0.05 were considered statistically significant.

**Results:**

The women not seeking CCS vs those seeking CCS had higher barriers according to Domain 1 of the CPC-28 (median; interquartile range: 33.33; 28.70–40.74 vs 14.82; 7.41–29.63; p<0.001). The risk of not seeking CCS was statistically significant in non-working (OR; 95 % CI: 2.458; 1.127–5.358; p<0.024), non-childbearing women (OR; 95 % CI: 3.302; 1.421–7.671; p<0.006) and women without cervical cancer (OR; 95 % CI: 4.709; 1.960–11.317; p<0.001).

**Conclusions:**

We identified barriers to having a Pap test in both of our groups. The risk of not seeking the CCS was statistically significant in non-working, non-childbearing women and women without cervical cancer.

## 1. Introduction

Slovakia is a country in Middle Europe belonging to a group of developed countries with well-organized healthcare systems that include free preventive gynecological examinations with cervical cancer screening. On average, 650 new cervical cancer cases occur in Slovakia each year, especially among women of productive age [1]. According to available data from health insurance companies, only 46% of women participate in preventive gynecological examinations. In comparison with other countries this is a very low percentage [2]. Therefore, it is necessary to understand and define the reasons why women do not attend preventive gynecological check-ups. A barrier to screening is a specific attitude, opinion or state that prevents the patient from seeking preventive care. The barriers to cervical cancer screening, according to the results of numerous studies, can be divided into five main groups: informational; psychological; socio-economic; behavioral and cultural; and geographical [3]. The boundaries between the groups of barriers are blurred and overlap each other. Reasons why women do not participate in screening are subjective and therefore difficult to clearly describe and define [4-6]. In Slovakia we have not found any studies aimed at the identification of barriers to cervical cancer prevention. In countries with much higher cervical cancer screening utilization (US, Sweden, Norway, Italy), many scientific studies are aimed at a better understanding of the barriers among women who do not participate in the preventive program [7]. Irregular or no participation in the screening examination is connected to the diagnosis of cervical intraepithelial lesions in advanced stages. Non-participation in the screening program is considered to be one of the risk factors for further development of cervical cancer [8-10]. The aim of this study was to identify and compare barriers to cervical cancer screening (CCS) between women seeking and not seeking CCS through the use of the CPC-28 questionnaire (the CPC-28 questionnaire: ‘Creencias, Papanicolaou, Cancer-28’ questionnaire – Beliefs about Papanicolaou and Cervical Cancer).

## 2. Methods

### 2.1 Study Design / Data Collection

**Figure 1:**
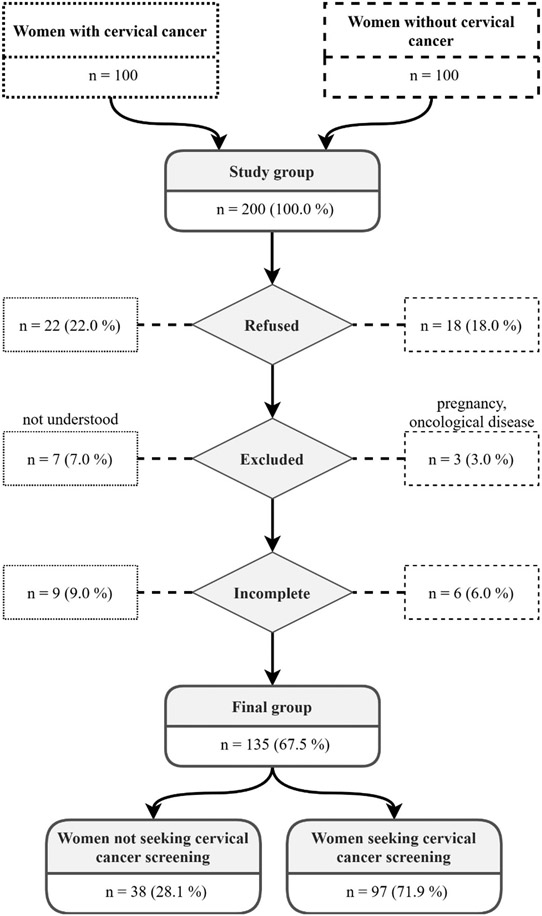
Study Flow Chart.

Two hundred women were included in the study because 30–40% of the population was expected to decline according to the inclusion and exclusion criteria. Of the 62 gynecological outpatient departments in Northern Slovakia, 20 departments were randomly involved in the cross-sectional comparative study. Random sampling according to MS Office Excel 2016 was used for simple randomization (Figure 1). Each department sent the CPC-28 questionnaire – to five healthy women and five women with untreated cervical cancer. Inclusion criteria were: over 23 years of age [11], no oncological disease (for the healthy women) and diagnosed with previously untreated cervical cancer (for the women with untreated cervical cancer). Exclusion criteria were: pregnancy; other oncological disease; impaired cognitive functions; incomplete questionnaire; and refusal to participate (questionnaire not completed). Women included (135/200) and excluded from the study are shown in Figure 1.

### 2.2 Questionnaire

A validated and standardized questionnaire was used as the instrument for data collection: the CPC-28 questionnaire. The source was an original questionnaire developed and validated in 2009 (Cronbach’s α = 0.735). The importance of this study was to develop a questionnaire to address the five health belief model components (severity, susceptibility, benefits, barriers, and cues to action) [12]. We translated and validated the CPC-28 questionnaire into the Slovak language (Cronbach’s α > 0.8 in all six domains) [13]. In the introductory part, the questions are aimed at demographic indicators, gynecological history and the presence or absence of chronic diseases and other cervical cancer risk factors [9]. The CPC-28 questionnaire consists of 28 statements [12,13]. Women indicate one of the four alternatives provided to show whether they agree or disagree with the given sentence. The statements are divided into six domains (Domain 1: Barriers to having a Pap test; Domain 2: Cues to action to having a Pap test; Domain 3: Severity of cervical cancer; Domain 4: Need to have a Pap test; Domain 5: Susceptibility to cervical cancer; Domain 6: Benefit to having a Pap test); nine questions aimed at the barriers to cervical cancer screening are in Domain 1 (Table 1). The labelling of the statements in this article comes from the order in the original version of the questionnaire. In the case of agreement with the given sentence, a barrier is present. A four-point Likert scale was used to assess responses (1: Strongly agree, 2: Agree, 3: Disagree, 4: Strongly disagree). To each answer the corresponding item score was added. A linear transformation was made to calculate the responses for a range of 0–100, according to the formula adjusted to each domain. The higher the score on the scale from 0–100, the stronger the barrier.

### 2.3 Statistical Analysis

Analysis and statistical evaluation of data were made using the computer programs Microsoft Office Excel 2007, Epi Info™ 7.1.5 and Statistica 13. The data from the introductory part of the questionnaire (demography, gynecological history, risk factors) were analyzed by using descriptive statistics. Chi-square test, non-parametric tests (Mann–Whitney U test) and odds ratio were used for statistical significance rating. Differences with a p value of <0.05 were considered statistically significant. The study was approved by the Ethics Committee of the Jessenius Faculty of Medicine Comenius University under the protocol number EK1431/13. Informed consent was obtained from all individual participants included in the study.

## 3. Results

The participants were divided into women not seeking CCS (8 healthy women vs 30 women with cervical cancer) and women seeking CCS (54 healthy women vs 43 women with cervical cancer). The median age was 32.0 years (interquartile range 23–48 years) in the women not seeking CCS vs 37.0 (interquartile range 30–47 years) in those seeking CCS. There were no statistically significant differences between the two groups (p = 0.189). Descriptive statistics for the file are shown in Table 2. To evaluate the results, the quantification of the barrier was determined based on the value of the scale. A higher value range indicated a stronger barrier. On comparing the range for Domain 1 between the women not seeking and those seeking CCS (median; interquartile range: 33.33; 28.70–40.74 vs 14.82; 7.41–29.63) there were statistically significant differences (p<0.001). The statistically significant differences were found in Domains 4 and 6 (Table 3). The results in Domains 4 and 6 confirmed the results of Domain 1 on the presence of barriers in women not seeking cervical cancer screening. Cervical cancer screening barriers were investigated in Domain 1. The statistically significant differences between the women not seeking and seeking CCS were found in all items of Domain 1 (Table 4). The odds ratio of not seeking CCS was statistically significant in the non-working, non-childbearing women and the women without cervical cancer. Education, smoking, hormonal contraception, and chronic diseases also increased the risks of not seeking CCS but were not statistically significant. This indicates the influence of risk factors that limit seeking CCS (Table 5).

## 4. Discussion

Current data show that the women’s reasons for non-attendance at screening are diverse [14-18]. Van der Meij et al identified the benefits and barriers that are important for women's decision-making about screening. According to these findings, they suggested improving the content of leaflets aimed at supporting women's decision-making. They found a number of differences in perceptions of benefits and harms between women with lower numeracy/health literacy and women with higher numeracy/literacy [19]. In all items of Domain 1 of the CPC-28 questionnaire (Barriers to having a Pap test), we found higher scores in the women not seeking CCS. This means that women had little information about the age for the first Pap test and its frequency, had problems in getting an appointment, or had to wait a long time for the test, did not have enough time to go for the Pap test, were afraid of cancer screening or detection. Namely, the risk of not seeking the CCS was statistically significant in non-working, non-childbearing women and women without cervical cancer. There was an interesting study in which the authors addressed the question of cervical cancer screening utilization among women living in England (London), who were originally from Slovakia, Poland and Romania. The women were informed about cervical cancer screening but they did not understand its importance for their health. They said they were positively motivated by invitation letters and reminders; however, they did not know how often the Pap test was performed and at what age it was necessary to undergo it for the first time [20]. In our research, 38.7% of women did not have enough information about the age for a Pap test (item A4), while 36.1% subjects in the women seeking CCS and 39.4% subjects in the women not seeking CCS did not have knowledge about the frequency of cervical cancer screening. A study conducted in Germany and Norway found that married women, mothers and non-smokers underwent the Pap test more often than unmarried women. In these countries, women receive a reminder every three years to undergo a screening. Women who underwent the screening had better knowledge about its frequency and screening attendance increased with age [21]. Long waiting times for the check-up (item A5) were considered to be a barrier for 21.1% of the women not seeking CCS, with a statistically significant difference in relation to the second group where this barrier was present in only 7.3% of women. Compared with studies from other countries, we have found more time-related barriers in Slovakia. In Poland, research was carried out at secondary schools and universities in Krakow that involved 400 women aged 17–26 years; overall, 11.2% of women perceived screening as time-consuming [22]. Inadequate behavior of healthcare workers (item A3) was a statistically significant stronger barrier present in 2.6% of the women not seeking CCS vs 1.0% of women seeking CCS. Women in England described disappointment with the doctor’s approach and felt better if they perceived empathy and a more sensitive attitude [23]. Compared to other countries, our research found far weaker barriers related to inadequate or inappropriate behavior of medical staff and healthcare workers (doctors or nurses). Fear of positive cervical cancer diagnosis (item A7) was seen as a barrier in 2.1% of the women seeking CCS vs 0.0% of the women not seeking CCS. Embarrassment at undergoing a gynecological examination (item A9) was present in 5.2% of the women seeking CCS and in 2.6% of the women not seeking it. In the Danish study, embarrassment (16.6%) and fear and anxiety (8.4%) decreased with age. An unpleasant experience from previous genital examinations increased with age. Pregnancy, breastfeeding or infertility treatment was a reason for not participating in screening. Interestingly, 0.7% of women do not participate in screening purely on principle, through their own convictions, without specifying further reasons. However, Denmark is one of several countries where experience has confirmed that invitations directly from a doctor lead to a slight increase in screening participation and willingness to undergo the Pap test [24]. In the women seeking CCS and the women not seeking CCS, 7.2% and 13.1% of women, respectively, did not have enough time to undergo cervical cancer screening (item A2). Opening hours of healthcare or gynecological centers were not compliant with women’s time management (item A8) in 2.0% and 10.5% of the women seeking CCS and not seeking CCS, respectively. Problems with getting an appointment for the preventive gynecological screening (item A11) were expressed as a barrier in 5.1% and 13.1% of the women seeking CCS and not seeking CCS, respectively. In Denmark, 32.3% of the 9484 women participating in the study reported organizational barriers as the main reason for not participating in regular screening. Most often they had forgotten to keep an appointment. Other problems with appointments were seen in 9.8% of women [24]. In healthcare systems where it is necessary to make an appointment for an examination, appointment difficulties can pose a significant barrier to cervical cancer screening. The psychological barriers involved in the CPC-28 questionnaire include the fear of positive examination results and embarrassment. In Chile, 127 women diagnosed with cervical cancer were involved in the study. This group of women is much more sensitive to cervical cancer-related issues and barriers to cervical cancer screening were found in 38%, including embarrassment (50%), inadequate behavior of healthcare workers and a negative experience from previous examinations preventing their return. Time-related problems, fear of diagnosis and lack of knowledge about the preventive effect of the Pap test were expressed as other barriers [25,26]. There is a lack of randomized controlled trials designed to specifically address falling cervical screening uptake amongst young women [27]. Educating women about barriers and training the healthcare professionals can facilitate an effective dialogue between two groups [28,29].

## 5. Conclusions

In conclusion, after evaluating the barriers to cervical cancer screening and the comparisons made between both of our groups, we can conclude that the existence of the barriers is statistically significant for some demographic selections. Namely, the risk of not seeking the CCS was statistically significant in non-working, non-childbearing women and women without cervical cancer. On the basis of these results, it will be possible to propose a change in the way preventive health services are offered and to address target populations in efforts to improve the quality of public health. It is important to minimize time barriers. It is recommended to educate healthcare workers about appropriate communication with women during preventive examinations. In Slovakia, in 2019, the Ministry of Health introduced screening invitation letters as part of a state-controlled screening program.

### Strengths and Limitations

This research is the first of its kind in Slovakia and can serve as a source for similar research in relation to other preventive examinations and screening programs. Our results were realized by a standardized and validated CPC-28 questionnaire. Comparing our results with those of other countries will help to understand the barriers to cervical cancer screening. When comparing our results with the results of studies in other countries it is important to understand the different socio-economic and cultural conditions, the demographic characteristics of women’s populations, the various methodologies applied and the target groups of women in the research. The limitation of our study is in the local evaluation of the women’s population. On the other hand, it is positive that the issues related to cervical cancer screening are studied and compared globally. The results of such studies may contribute to a better understanding of the reasons why people do not care about their health as they could.

## Figures and Tables

**Table 1:** The statements in CPC-28 aimed at the identification of cervical cancer screening barriers – Domain 1^a^.

Number	Text
A2	I do not have time to get a Pap test.
A3	I have not taken the Pap test because they treat me badly in the healthcare centre.
A4	I do not know at what age it is necessary to have a Pap test.
A5	I have not taken a Pap test because when I go, I need to wait a long time to be seen.
A7	I have not taken the Pap test because I am afraid to find out if I have cancer.
A8	I have not taken the Pap test because the healthcare centre is only open during hours when I cannot go.
A9	I have not taken the Pap test because I am embarrassed to have a genital examination.
A10	I do not know how often I need to get a Pap test.
A11	I have not taken a Pap test because it is difficult to get an appointment.

a Domain 1, Barriers to having a Pap test.

**Abbreviation:** CPC-28- CPC-28 questionnaire (‘Creencias, Papanicolaou, Cancer-28’ questionnaire – Beliefs about Papanicolaou and Cervical Cancer).

**Table 2:** The basic characteristics of study participants (N=135).

Characteristics	No	Yes
	N (%)	N (%)
Preventive examination	38 (28.1)	97 (71.9)
University education	74 (54.8)	61 (45.2)
Working women	44 (32.6)	91 (67.4)
Number of childbirths: 1 or more	31 (23.0)	104 (77.0)
Smokers	107 (79.3)	28 (20.7)
Taking HC	84 (62.6)	51 (37.8)
With cervical cancer	73 (54.1)	62 (45.9)
With chronic disease’s	56 (41.5)	79 (58.5)

**Abbreviation:** HC- Hormonal Contraception.

**Table 3:** Domains scale comparison (CPC-28) in the women seeking and not seeking cervical cancer screening.

Domain	Women seeking CCS (N=97)	Women not seeking CCS (N=38)	p value*
	median (interquartile range)	median (interquartile range)	
1	14.82 (7.41 - 29.63)	33.33 (28.70 - 40.74)	< 0.001
2	44.44 (33.33 - 61.11)	44.44 (33.33 - 61.11)	0.879
3	16.68 (0.00 - 33.33)	25.00 (0.00 - 33.33)	0.416
4	22.22 (0.00 - 33.33)	33.33 (19.44 - 36.11)	0.049
5	33.33 (33.33 - 44.44)	44.44 (33.33 - 55.56)	0.23
6	0.00 (0.00 - 22.22)	22.22 (8.33 - 25.00)	0.003

* Mann-Whitney U test.

**Abbreviation:** CPC-28- CPC-28 questionnaire (‘Creencias, Papanicolaou, Cancer-28’ questionnaire – Beliefs about Papanicolaou and Cervical Cancer); CCS- Cervical Cancer Screening.

**Table 4:** Domain 1^a^: comparison of the responses in the women seeking and not seeking CCS.

	Women seeking CCS (N=97)				Women not seeking CCS (N=38)				
	N (%)				N (%)				
Item	Strongly agree	Agree	Disagree	Strongly disagree	Strongly agree	Agree	Disagree	Strongly disagree	p value*
A2	1 (1.0)	6 (6.2)	34 (35.1)	56 (57.7)	1 (2.6)	4 (10.5)	23 (60.5)	10 (26.3)	0.012
A3	1 (1.0)	0 (0.0)	36 (37.1)	60 (61.9)	0 (0.0)	1 (2.6)	26 (68.4)	11 (28.9)	0.002
A4	6 (6.2)	29 (29.9)	28 (28.9)	34 (35.1)	4 (10.5)	11 (28.9)	20 (52.6)	3 (7.9)	0.006
A5	2 (2.1)	5 (5.2)	40 (41.2)	50 (51.5)	2 (5.3)	6 (15.8)	21 (55.3)	9 (23.7)	0.013
A7	0 (0.0)	2 (2.1)	40 (41.2)	55 (56.7)	0 (0.0)	0 (0.0)	25 (65.8)	13 (34.2)	0.031
A8	1 (1.0)	1 (1.0)	34 (35.1)	61 (62.9)	1 (2.6)	3 (7.9)	26 (68.4)	8 (21.1)	< 0.001
A9	2 (2.1)	3 (3.1)	34 (35.1)	58 (59.8)	0 (0.0)	1 (2.6)	26 (68.4)	11 (28.8)	0.03
A10	5 (5.2)	25 (25.8)	34 (35.1)	33 (34.0)	4 (10.5)	12 (31.6)	18 (47.4)	4 (10.5)	0.044
A11	1 (1.0)	4 (4.1)	40 (41.2)	52 (53.6)	4 (10.5)	1 (2.6)	28 (73.7)	5 (13.2)	< 0.001

* Chi-square test.

a Domain 1, Barriers to having a Pap test.

**Abbreviation:** CCS- Cervical Cancer Screening.

**Table 5:** The Odds ratio to cervical cancer screening in the women not seeking CCS.

	Odds ratio	95 % CI	p value
University education	1.76	0.826 - 3.751	0.143
Not working women^a^	2.458	1.127 - 5.358	0.024
Number of childbirths: 0	3.302	1.421 - 7.671	0.006
Non-smokers	2.051	0.717 - 5.865	0.18
Women not taking HC	1.723	0.767 - 3.869	0.188
Women without cervical cancer	4.709	1.960 - 11.317	< 0.001
Women with chronic disease’s	2.124	0.948 - 4.759	0.067

a Unemployed, student, maternity leave, retired.

**Abbreviation:** CCS- Cervical Cancer Screening; CI- Confidence Interval; HC- Hormonal Contraception.

## Data Availability

The datasets used and analyzed during the current study are available from the corresponding author on reasonable request.

## Abbreviations:

CCS: Cervical Cancer Screening
CPC-28 Questionnaire: ‘Creencias, Papanicolaou, Cancer-28’ questionnaire (Beliefs about Papanicolaou and Cervical Cancer)
Pap Test: Papanicolaou Test
